# 8-Cl-Adenosine enhances 1,25-dihydroxyvitamin D_3_-induced growth inhibition without affecting 1,25-dihydroxyvitamin D_3_-stimulated differentiation of primary mouse epidermal keratinocytes

**DOI:** 10.1186/1471-2210-4-13

**Published:** 2004-07-27

**Authors:** Wendy B Bollag, Xiaofeng Zhong, Sarah Josephson

**Affiliations:** 1 Department of Medicine (Dermatology), Medical College of Georgia, Augusta, GA 30912 USA; 2 Cell Biology and Anatomy, Medical College of Georgia, Augusta, GA 30912 USA; 3 Institute of Molecular Medicine & Genetics, Medical College of Georgia, Augusta, GA 30912 USA

## Abstract

**Background:**

Epidermal keratinocytes continuously proliferate and differentiate to form the mechanical and water permeability barrier that makes terrestrial life possible. In certain skin diseases, these processes become dysregulated, resulting in abnormal barrier formation. In particular, skin diseases such as psoriasis, actinic keratosis and basal and squamous cell carcinomas are characterized by hyperproliferation and aberrant or absent differentiation of epidermal keratinocytes. We previously demonstrated that 8-Cl-adenosine (8-Cl-Ado) can induce keratinocyte growth arrest without inducing differentiation.

**Results:**

To determine if this agent might be useful in treating hyperproliferative skin disorders, we investigated whether 8-Cl-Ado could enhance the ability of 1,25-dihydroxyvitamin D_3 _[1,25(OH)_2_D_3_], a known keratinocyte differentiating agent and a clinical treatment for psoriasis, to inhibit keratinocyte growth. We found that low concentrations of 8-Cl-Ado and 1,25(OH)_2_D_3 _appeared to act additively to reduce proliferation of primary mouse epidermal keratinocytes. However, another agent (transforming growth factor-beta) that triggers growth arrest without inducing differentiation also coincidentally inhibits differentiation elicited by other agents; inhibition of differentiation is suboptimal for treating skin disorders, as differentiation is often already reduced. Thus, we determined whether 8-Cl-Ado also decreased keratinocyte differentiation induced by 1,25(OH)_2_D_3_, as measured using the early and late differentiation markers, keratin 1 protein levels and transglutaminase activity, respectively. 8-Cl-Ado did not affect 1,25(OH)_2_D_3_-stimulated keratin 1 protein expression or transglutaminase activity.

**Conclusions:**

Our results suggest that 8-Cl-Ado might be useful in combination with differentiating agents for the treatment of hyperproliferative disorders of the skin.

## Background

The epidermis of the skin serves as a mechanical and water permeability barrier essential for terrestrial life (reviewed in [1]) and is composed primarily of epidermal keratinocytes. These keratinocytes stratify to form several layers. The deepest layer, the stratum basalis or basal layer comprises proliferating cells that continuously divide to regenerate cells lost to the environment. As the cells migrate upward into the first differentiated layer, the stratum spinousum or spinous layer, they cease proliferating and begin to express the intermediate filament proteins, the mature keratins 1 and 10. This early differentiation is followed by a late differentiation program in the stratum granulosum or granular layer, which is marked by the expression of other structural proteins, such as filaggrin and loricrin, and by increased activity of the enzyme, transglutaminase, which forms highly durable γ-glutamyl-ε-lysyl bonds to cross-link the proteins into a tough and resistant shell underneath the plasma membrane. At the boundary of the granular layer and the outermost stratum corneum, or cornified layer, the keratinocytes terminally differentiate, degrading their nuclei and other organelles and releasing lamellar bodies, the lipid contents of which form a water-impermeant barrier. The squames, the flattened remnants of the keratinocytes, and the lipids from the lamellar bodies form a sort of brick and mortar, to prevent water loss, microbial invasion and/or other mechanical insults (reviewed in [2-4]).

1,25-Dihydroxyvitamin D_3 _[1,25(OH)_2_D_3_] is a known regulator of this process of keratinocyte growth and differentiation (reviewed in [2,5]). *In vitro*, 1,25(OH)_2_D_3 _inhibits keratinocyte proliferation and stimulates the expression of numerous keratinocyte differentiation markers (reviewed in [2,6]). *In vivo *a physiologic role for 1,25(OH)_2_D_3 _in regulating keratinocyte differentiation is suggested by several lines of evidence: (1) keratinocytes express both the 25-hydroxylase and the 1α-hydroxylase which converts inactive vitamin D_3 _to its active 1,25-dihydroxy metabolite (reviewed in [2,6]); (2) receptors for 1,25(OH)_2_D_3 _are present in the skin and in epidermal keratinocytes *in vitro *[7-11]; and (3) Vitamin D receptor null mice exhibit altered skin function, characterized by abnormal hair follicles and reduced expression of several keratinocyte differentiation markers [12]. Furthermore, 1,25(OH)_2_D_3 _and its structural analogs have been used as effective treatments for psoriasis, a human skin disease characterized by inflammation and by hyperproliferation and abnormal differentiation of keratinocytes (reviewed in [13,14]).

8-Chloro-cyclic-adenosine monophosphate (8-Cl-cAMP) is known to inhibit growth and to induce apoptosis in a variety of cancer cells [15-18], suggesting its potential utility as an anti-cancer drug. Indeed, phase I trials with 8-Cl-cAMP have been performed ([19,20] and reviewed in [21]) and phase II trials are in progress [22]. However, the mechanisms by which this agent acts are incompletely understood, and several investigators have proposed that an 8-Cl-cAMP metabolite, 8-chloro-adenosine (8-Cl-Ado) is the active anti-proliferative compound [16,23]. Indeed, 8-Cl-Ado has been shown to inhibit growth in a variety of cell types [24-28].

Previously, we demonstrated that 8-Cl-Ado arrests the growth of primary mouse epidermal keratinocytes without triggering differentiation [29]. Thus, 8-Cl-Ado functions in an analogous fashion to transforming growth factor-β (TGF-β), which also triggers growth arrest, but not differentiation,, of keratinocytes (reviewed in [30]). In contrast with a polypeptide such as TGF-β, 8-Cl-Ado, as a small molecule rather than a protein, could potentially be taken orally or applied topically to skin. Thus, 8-Cl-Ado may represent a novel therapy for treatment of skin disorders, such as psoriasis, actinic keratoses and basal and squamous cell carcinomas, characterized by hyperproliferation of keratinocytes. One potential problem, however, is that TGF-β also inhibits the expression of differentiation markers elicited by other differentiating agents [31]. Since another characteristic typical of hyperproliferative skin diseases such as psoriasis is impaired differentiation [32], a therapy that inhibits both proliferation and differentiation would be less than ideal.

To determine whether 8-Cl-Ado, as a potent keratinocyte growth arrestor, could potentially be used to treat hyperproliferative skin diseases in combination with a current treatment, we investigated the effect of 8-Cl-Ado on 1,25(OH)_2_D_3_-induced inhibition of keratinocyte proliferation and stimulation of keratinocyte differentiation. We found that low concentrations of 8-Cl-Ado acted additively with 1,25(OH)_2_D_3 _to inhibit DNA synthesis, without affecting the ability of 1,25(OH)_2_D_3 _to enhance keratin 1 expression, a marker of early differentiation, or transglutaminase activity, a marker of late differentiation. Thus, our results suggest that a combination therapy with 1,25(OH)_2_D_3 _and 8-Cl-Ado could potentially be an effective treatment for hyperproliferative skin disorders including psoriasis, actinic keratosis and non-melanoma skin cancers.

## Results and discussion

**Figure 1:**
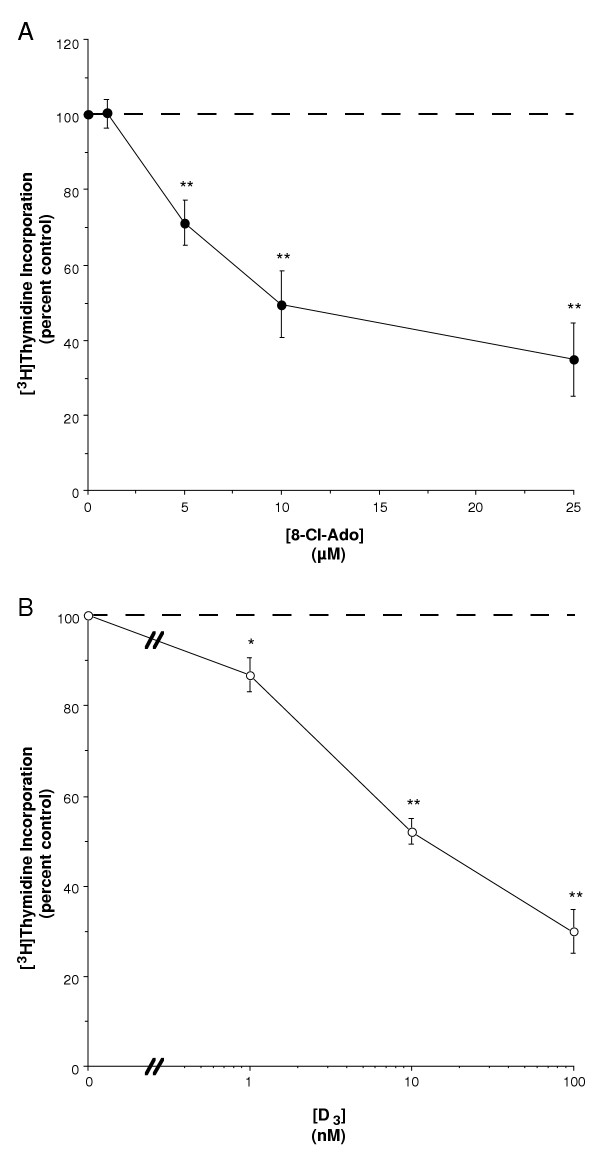
**8-Cl-Ado and 1,25(OH)_2_D_3 _Inhibit Keratinocyte Proliferation. **Near-confluent primary mouse epidermal keratinocytes were treated with the indicated concentrations of (A) 8-Cl-Ado or (B) 1,25(OH)_2_D_3 _for 24 hours, and [^3^H]thymidine incorporation was determined as indicated in Materials and Methods. Data represent the mean ± SEM of five experimentsperformed in triplicate; *p < 0.05, **p < 0.01 versus the control value.

**Figure 2:**
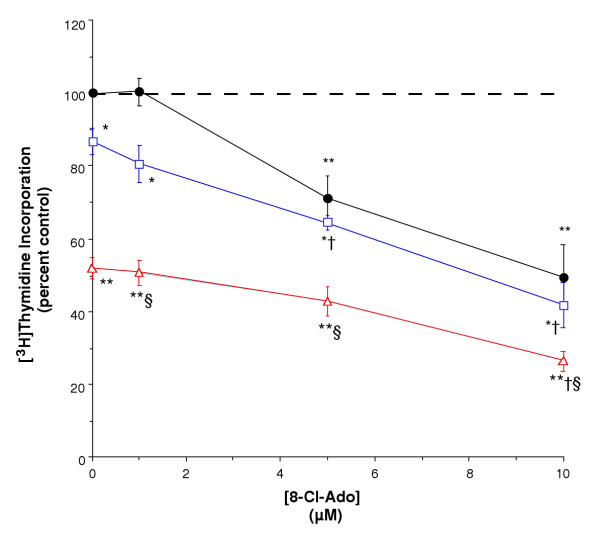
**8-Cl-Ado and 1,25(OH)_2_D_3 _Act Additively to Inhibit Keratinocyte Proliferation. **Near-confluent primary mouse epidermal keratinocytes were treated with the indicated concentrations of 8-Cl-Ado in the presence of no (closed circles), 1 nM (open squares) or 10 nM (open triangles) 1,25(OH)_2_D_3 _for 24 hours, and [^3^H]thymidine incorporation was determined as indicated in Materials and Methods. Data represent the mean ± SEM of five experiments performed in triplicate; *p < 0.05, **p < 0.01 versus the control value, †p < 0.01 versus the corresponding concentration of 1,25(OH)_2_D_3 _alone, §p < 0.01 versus the corresponding concentration of 8-Cl-Ado alone.

To determine if 8-Cl-Ado could function with the growth inhibiting agent 1,25(OH)_2_D_3 _to enhance its antiproliferative effect, we incubated primary epidermal keratinocytes for 24 hours with various concentrations of 8-Cl-Ado in the presence and absence of low concentrations of 1,25(OH)_2_D_3 _prior to assessing effects on de novo DNA synthesis as measured by [^3^H]thymidine incorporation into DNA. As shown in Figure 1A, 8-Cl-Ado inhibited [^3^H]thymidine incorporation at concentrations of 5–25 μM with an estimated half-maximal inhibitory concentration (IC_50_) of 5 μM. This value agrees well with our previously determined IC_50 _of 7.5 μM [29]. In agreement with previous reports [33,34], 1,25(OH)_2_D_3 _also inhibited DNA synthesis at concentrations of 1 to 100 nM with an estimated IC_50 _of approximately 4 nM (Figure 1B). As shown in Figure 2, when the two agents were combined, their effect on DNA synthesis appeared to be additive, as evidenced by the comparable slopes of the [^3^H]thymidine incorporation curves at the three different concentrations of 0 (a portion of which is replotted from Figure 1), 1 and 10 nM 1,25(OH)_2_D_3_. The combination of 1 or 5 μM 8-Cl-Ado with 10 nM 1,25(OH)_2_D_3 _yielded a greater inhibition than 8-Cl-Ado alone, and conversely, the combined effect of 5 and 10 μM 8-Cl-Ado with 1 nM 1,25(OH) _2_D_3 _was significantly larger than 1 nM 1,25(OH)_2_D_3 _alone. Importantly, the combination of 10 nM 1,25(OH)_2_D_3 _with 10 μM 8-Cl-Ado produced an inhibition of [^3^H]thymidine incorporation that was significantly greater than that elicited by either agent alone. Indeed, the inhibition elicited by 10 μM 8-Cl-Ado and 10 nM 1,25(OH)_2_D_3 _was comparable to the inhibition produced by 100 nM 1,25(OH)_2_D_3 _alone (compare Figures 1B and 2). Thus, our results suggest that not only does 8-Cl-Ado not prevent the growth inhibitory action of 1,25(OH)_2_D_3_, but, in fact, the two agents seem to act in an additive fashion to more effectively inhibit keratinocyte proliferation.

**Figure 3:**
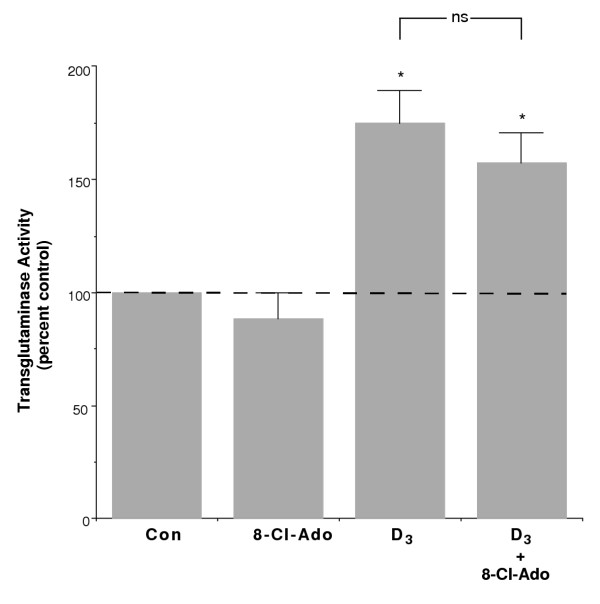
**8-Cl-Ado Has No Effect on 1,25(OH)_2_D_3_-Stimulated Transglutaminase Activity. **Near-confluent primary mouse epidermal keratinocytes were treated with and without 10 μM 8-Cl-Ado in the presence and absence of 10 nM 1,25(OH)_2_D_3 _for 24 hours, and transglutaminase activity was determined as indicated in Materials and Methods. Data represent the mean ± SEM of four experiments performed in triplicate; *p < 0.01 versus the control value.

TGF-β, another agent that, like 8-Cl-Ado, induces growth arrest but not differentiation of keratinocytes ([31] and reviewed in [30]), can inhibit the ability of differentiating agents to elicit keratinocyte differentiation [31]. However, for an agent to have therapeutic potential as a treatment for hyperproliferative skin disorders, such an inhibition of differentiation would be counterproductive to its efficacy as a medication. To determine if 8-Cl-Ado also inhibited keratinocyte differentiation, we investigated whether 8-Cl-Ado inhibited the ability of 1,25(OH)_2_D_3 _to induce the late differentiation marker, transglutaminase activity. For this experiment we chose the concentrations of 8-Cl-Ado (10 μM) and 1,25(OH)_2_D_3 _(10 nM) shown in Figure 2 to produce a greater growth inhibition than either agent alone. As illustrated in Figure 3, 10 μM 8-Cl-Ado alone had little or no effect on transglutaminase activity, as reported previously [29]. On the other hand, 10 nM 1,25(OH)_2_D_3 _significantly elevated transglutaminase activity by approximately 75%. The combination of 8-Cl-Ado and 1,25(OH)_2_D_3_was not significantly different from 1,25(OH)_2_D_3 _alone, with a significant approximate 60% increase relative to the control value. Thus, our results indicate that 8-Cl-Ado did not prevent the differentiative effect of 1,25(OH)_2_D_3_, suggesting that these two agents might be combined to treat keratinocyte hyperproliferative disorders, such as psoriasis.

**Figure 4:**
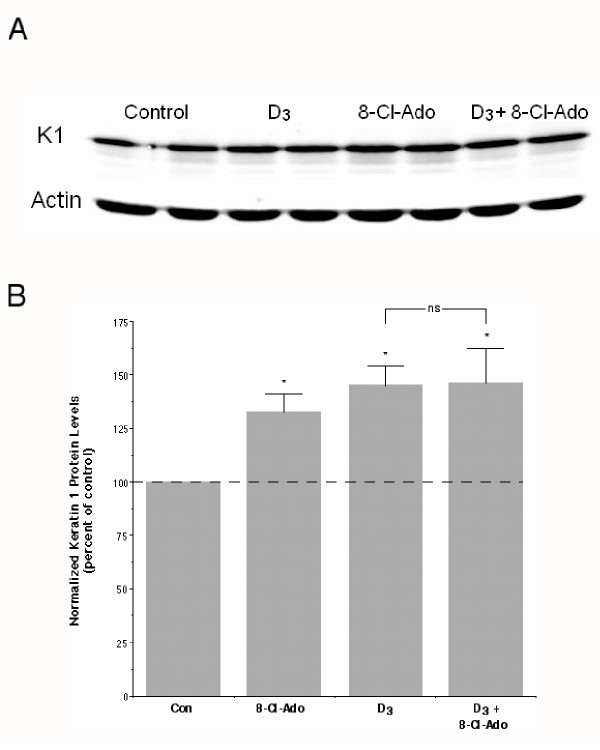
**8-Cl-Ado Has No Effect on the 1,25(OH)_2_D_3_-Induced Increase in Keratin 1 Protein Levels. **Near-confluent keratinocytes were incubated for 24 hours with and without 25 μM 8-Cl-Ado in the presence and absence of 20 nM 1,25(OH)_2_D_3 _and were then processed for western analysis. (A) A representative immunoblot is shown. (B) Keratin 1 levels were quantified, corrected for background and normalized for loading, as described in Materials and Methods. Data represent the mean ± SEM of four experiments performed in duplicate; *p < 0.05 versus the control value.

Transglutaminase activity is a marker of late keratinocyte differentiation. We also examined the effect of 8-Cl-Ado on a marker of early keratinocyte differentiation, namely keratin 1 protein expression, using an even higher concentration of 8-Cl-Ado (25 μM). Western analysis demonstrated that 1,25(OH)_2_D_3 _induced an approximate 45% increase in keratin 1 protein levels with the combination of 1,25(OH)_2_D_3 _and 8-Cl-Ado producing a comparable 46% increase (Figure 4). Thus, early differentiation in response to 1,25(OH)_2_D_3 _also was not affected by 8-Cl-Ado. Interestingly, however, in contrast to previous results [29], in these experiments 8-Cl-Ado alone elicited a small but significant increase in keratin 1 protein expression (32%). The reason for this disparity is unclear but may result from differences in the lot of anti-keratin 1 antibody used in the western analysis and/or the increased sensitivity of the method used for detecting and quantifying immunoreactive protein in this work.

Most current treatments for psoriasis suffer from one or more disadvantages including lack of efficacy, contraindications due to deleterious side effects and/or aesthetic deficiencies ([35] and reviewed in [36]). Indeed, monotherapies tend to be less efficacious than combination therapies with two or more agents used concurrently, sequentially or in a rotational fashion (reviewed in [36]). Treatment with 1,25(OH)_2_D_3 _and its analogs has proven successful, although the possibility of toxicity as the result of 1,25(OH)_2_D_3_'s ability to affect calcium metabolism has led to the search for topically effective analogs with little or no effect on serum calcium levels (reviewed in [32]). If the amount of 1,25(OH)_2_D_3 _(or its analog) required for treatment could be reduced, this decrease in dosage would presumably minimize systemic effects on calcium, which is the primary dose-limiting factor in the use of 1,25(OH)_2_D_3 _analogs in the treatment of psoriasis [32]. Thus, our results indicating that 8-Cl-Ado enhances the growth inhibitory effect of 1,25(OH)_2_D_3_, a known keratinocyte differentiating agent and possible treatment for psoriasis [32], suggests the potential for combination therapy. Moreover, the fact that 8-Cl-Ado does not interfere with the promotion of differentiation by 1,25(OH)_2_D_3 _further supports the possible combined use of these two agents for treatment of hyperproliferative skin disorders.

Several lines of evidence suggest that 8-Cl-Ado is not simply acting through cyototoxicity to inhibit keratinocyte growth. First, we have previously shown that 8-Cl-Ado growth arrests keratinocytes in the G_0_/G_1 _phase of the cell cycle with no increase in the sub-G_0_/G_1 _(apoptotic) population of cells [29]. Second, we also showed that the effect of 8-Cl-Ado to inhibit proliferation is reversible in that washout of the compound returned DNA synthesis essentially to basal (untreated) levels [29]. Finally, in this report we demonstrate that 8-Cl-Ado did not inhibit the 1,25(OH)_2_D_3_-stimulated increase in transglutaminase activity (Figure 3) or keratin 1 protein expression (Figure 4). Together, these results indicate that 8-Cl-Ado is acting in a specific manner to decrease keratinocyte proliferation.

Nevertheless, the mechanism by which 8-Cl-Ado exerts its growth inhibitory effects in keratinocytes is not clear. Our previous results indicate that 8-Cl-Ado must enter the cells to trigger growth arrest, since inhibiting uptake with an adenosine transporter, NBTI, prevented the arrest in the G_0_/G_1 _phase of the cell cycle [29]. We also reported in a prior publication that 8-Cl-Ado induced the expression of the cyclin-dependent kinase inhibitor, p21 [29], which is known to contribute to growth arrest in keratinocytes and other cell types ([37] and reviewed in [30]). However, other investigators have reported 8-Cl-Ado-mediated inhibitory effects on RNA synthesis and the levels of cellular ATP [16]. Clearly, further research is necessary to define the pathways used by 8-Cl-Ado to regulate keratinocyte proliferation.

## Conclusions

In summary, our data show that 8-Cl-Ado functions with the keratinocyte-differentiating agent 1,25(OH)_2_D_3 _to inhibit keratinocyte proliferation without altering the ability of 1,25(OH)_2_D_3 _to induce differentiation. Thus, our results support the possibility of using 8-Cl-Ado alone or in combination with differentiating agents such as 1,25(OH)_2_D_3 _or its analogs to treat hyperproliferative keratinocyte disorders including psoriasis.

## Methods

### Materials

Tissue culture reagents were obtained from standard suppliers as indicated in a previous publication [29]. 1,25(OH)_2_D_3 _was a generous gift of Dr. Maurice Pechet (Research Institute for Medicine and Chemistry, Cambridge, MA). 8-Cl-Ado was obtained from Biolog (La Jolla, CA). [^3^H]Thymidine and [^3^H]putrescine were purchased from Dupont/NEN (Boston, MA). Dimethylated casein was obtained from Sigma (St. Louis, MO). All other reagents were from standard suppliers.

### Keratinocyte culture

Primary cultures of mouse epidermal keratinocytes were prepared from neonatal ICR CD-1 mice and cultivated in a 25 μM calcium-containing serum-free keratinocyte medium as in [29].

### Measurement of DNA synthesis

For measurement of [^3^H]thymidine incorporation into DNA, as in [29], near-confluent cultures were refed with SFKM containing various concentrations of 8-Cl-Ado with or without different concentrations of 1,25(OH)_2_D_3_. After 24 hours, cells were labeled with 1 μCi/ml [^3^H]thymidine for an additional hour in the continued presence of 8-Cl-Ado and/or 1,25(OH)_2_D_3_. Cultures were washed twice with phosphate-buffered saline without calcium or magnesium (PBS^-^) and macromolecules were precipitated using ice-cold 5% trichloroacetic acid (TCA). After additional washing with 5% TCA and distilled water, cells were solubilized in 0.3 M NaOH, and the amount of [^3^H]thymidine incorporated into DNA was determined by liquid scintillation counting.

### Measurement of transglutaminase activity

Transglutaminase activity was assessed essentially as described in [33]. Briefly, near-confluent keratinocytes were incubated for 24 hours with the indicated agents in SFKM. The cells were scraped into homogenization buffer (0.1 M Tris-acetate, pH 7.8, 2 μg/ml aprotinin, 2 μM leupeptin, 1 μM pepstatin A, 0.2 mM EDTA and 0.2 mM PMSF), collected by centrifugation and subjected to one freeze-thaw cycle prior to disruption by sonication. Aliquots of the homogenate were removed for determination of protein content and transglutaminase activity. Transglutaminase activity was measured as the [^3^H]putrescine radioactivity incorporated into casein after an overnight incubation at 37°C. Casein was precipitated with TCA, collected onto glass fiber filters and counted by liquid scintillation spectrometry. The cellular protein content of the samples was determined using the Bio-Rad DC protein assay system (Bio-Rad, Hercules, CA), with BSA as standard, and transglutaminase activity was expressed as cpm/μg protein.

### Western analysis of keratin 1 protein levels

Keratinocytes were treated and solubilized in sample buffer (31.2 mM Tris, pH 6.8, 1% SDS, 12.5% glycerol). Equal sample volumes were separated by SDS polyacrylamide gel electrophoresis on an 8% gel and transferred to Immobilon PVDF membrane (Millipore, Billerica, MA). Membranes were blocked with Odyssey blocking buffer (Licor Biosciences, Lincoln, NE), probed with a rabbit polyclonal anti-keratin 1 antibody (Covance, Princeton, NJ) and a mouse monoclonal anti-actin antibody (Sigma, St. Loius, MO). Immunoreactive proteins were visualized with IRDye800-coupled donkey anti-rabbit IgG (Rockland Immunochemicals, Gilbertsville, PA) or IR Alexa Fluor 680-coupled goat anti-mouse IgG (Molecular Probes, Eugene, OR) on a Licor Odyssey Infrared Imaging System. Keratin-1 protein levels were corrected for background and normalized using background-corrected actin levels.

### Statistical analysis

Significance of differences was determined with the computer program InStat (Graphpad Software, San Diego, CA) using ANOVA with a Student-Newman-Keuls post-hoc test.

## Abbreviations

1,25(OH)_2_D_3_, 1,25-dihydroxyvitamin D_3_; 8-Cl-Ado, 8-chloro-adenosine; 8-Cl-cAMP, 8-chloro-cyclic-adenosine monophosphate; IC_50_, half-maximal inhibitory concentration; TGFβ, transforming growth factor-beta

## Authors' contributions

WBB conceived of the study, planned the experiments, analyzed the data and drafted the manuscript; XZ and SJ planned, conducted and analyzed the keratin 1 expression experiments.
